# Protective Gene Expression Changes Elicited by an Inherited Defect in Photoreceptor Structure

**DOI:** 10.1371/journal.pone.0031371

**Published:** 2012-02-20

**Authors:** Yagya V. Sharma, Radu I. Cojocaru, Linda M. Ritter, Nidhi Khattree, Matthew Brooks, Alison Scott, Anand Swaroop, Andrew F. X. Goldberg

**Affiliations:** 1 Eye Research Institute, Oakland University, Rochester, Michigan, United States of America; 2 Department of Ophthalmology and Visual Sciences, Kellogg Eye Center, University of Michigan, Ann Arbor, Michigan, United States of America; 3 Neurobiology Neurodegeneration & Repair Laboratory, National Eye Institute, National Institutes of Health, Bethesda, Maryland, United States of America; Dalhousie University, Canada

## Abstract

Inherited defects in retinal photoreceptor structure impair visual transduction, disrupt relationship with the retinal pigment epithelium (RPE), and compromise cell viability. A variety of progressive retinal degenerative diseases can result, and knowledge of disease etiology remains incomplete. To investigate pathogenic mechanisms in such instances, we have characterized rod photoreceptor and retinal gene expression changes in response to a defined insult to photoreceptor structure, using the *retinal degeneration slow* (*rds*) mouse model. Global gene expression profiling was performed on flow-sorted *rds* and wild-type rod photoreceptors immediately prior and subsequent to times at which OSs are normally elaborated. Dysregulated genes were identified via microarray hybridization, and selected candidates were validated using quantitative PCR analyses. Both the array and qPCR data revealed that gene expression changes were generally modest and dispersed amongst a variety of known functional networks. Although genes showing major (>5-fold) differential expression were identified in a few instances, nearly all displayed transient temporal profiles, returning to WT levels by postnatal day (P) 21. These observations suggest that major defects in photoreceptor cell structure may induce early homeostatic responses, which function in a protective manner to promote cell viability. We identified a single key gene, *Egr1*, that was dysregulated in a sustained fashion in *rds* rod photoreceptors and retina. *Egr1* upregulation was associated with microglial activation and migration into the outer retina at times subsequent to the major peak of photoreceptor cell death. Interestingly, this response was accompanied by neurotrophic factor upregulation. We hypothesize that activation of *Egr1* and neurotrophic factors may represent a protective immune mechanism which contributes to the characteristically slow retinal degeneration of the *rds* mouse model.

## Introduction

Human vision begins with rod and cone photoreceptors, light-sensitive ciliated sensory neurons situated in the neural retina. These fragile cells are susceptible to a variety of insults, which can impede their function and viability and cause retinal degeneration and vision loss. Vertebrate animal models, and in particular mice, have been a valuable resource to identify molecules essential for normal photoreceptor physiology. Although a wide variety of naturally occurring and engineered mouse models have been investigated for retinal degeneration [1], and a majority of vision loss in inherited photoreceptor degenerations is known to result from secondary pathogenic processes, the detailed mechanisms by which genetic defects cause retinal degeneration continue to be debated [2]–[5]. Further advances are needed to improve understanding of normal photoreceptor physiology and implement more effective clinical treatments. It is anticipated that insights into the relatively simple monogenic diseases can simultaneously shed light on widely prevalent loss-of-sight conditions with multifactorial etiologies.

The *retinal degeneration slow (rds)* mouse (also known as *Prph2^Rd2^*) has been the focus of numerous investigations of photoreceptor and retinal structure, function and viability [6]–[8]. This naturally occurring model results from a spontaneous insertion of viral DNA into the *rds* (also known as *Prph2*) gene to produce a null allele [9]. Complete loss of the gene product (peripherin/rds) in homozygous null animals prevents elaboration of rod and cone photoreceptor outer segments (OSs) – the specialized ciliary organelles upon which vertebrate light detection depends [10]. Surprisingly, this massive structural defect is not catastrophic for photoreceptor viability. Instead, cells undergo a relatively slow rate of degeneration that occurs over a period of months [11], [12], and has prompted evaluation of several therapeutic strategies [13]–[15].

A previous study applied a whole-retina microarray approach to the *rds* model to identify genes associated with retinal degeneration [16]. Here, we investigate the global transcriptome response of purified *rds* rod photoreceptors to lack of peripherin/rds and absence of OSs. Our findings suggest that a combination of homeostatic mechanisms may contribute to the protracted time course of retinal degeneration in the *rds* retina.

## Results

### Identification of differentially expressed genes in *rds* rod photoreceptors

**Figure 1:**
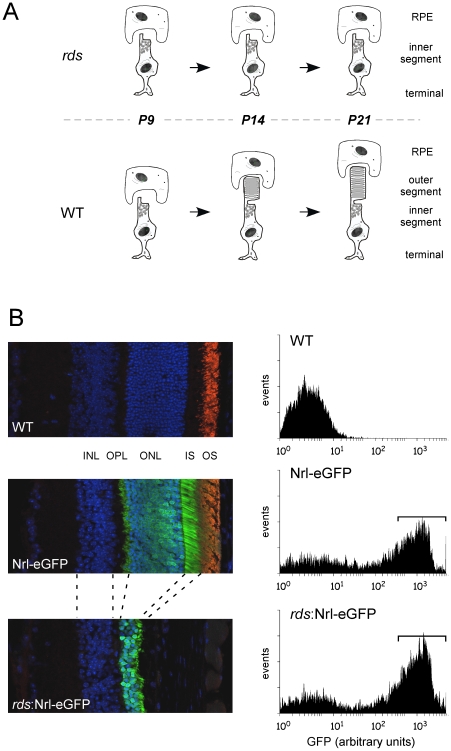
Strategy and sample generation for gene expression profiling of structurally abnormal rod photoreceptors. (A) Photoreceptors in the *rds* mouse fail to elaborate OSs. OS disk membrane biosynthesis begins at ∼P10, OSs are well established by P14, and reach full length by ∼P21. (B) *Left panels*: immunofluorescence analyses of retinal cryosections; DAPI-stained nuclei appear blue. eGFP labeled photoreceptors (green) were observed in WT, Nrl-eGFP and *rds*:Nrl-eGFP retinas; however, OSs labeled with anti-P/rds antibody PabMPCT (red), were only detected in WT and Nrl-eGFP retinas. *Right panels*: fluorescence-activated cell sorting was utilized to enrich rod photoreceptors from trypsin-dissociated mouse retinas. Typical histograms, generated using a BD Biosciences FACSVantage SE cell sorter, illustrate GFP-containing rod photoreceptors detected in dissociated (P9) retinas from Nrl-eGFP and *rds*:Nrl-eGFP, but not WT mice. Collected rod cell fractions, typically ∼500,000 cells per retina, are indicated (brackets).

To investigate how inherited defects in photoreceptor structure can affect cell viability, we adopted a flow-sorting method for identifying transcriptome changes in rod photoreceptors [17]. We applied this technique to the *rds* mouse retina, since this animal model possesses a well-defined monogenic defect in photoreceptor structure, and mutations in *rds* produce a broad spectrum of human retinal disease [18], [19]. Figure 1A illustrates the rationale underlying our approach. OSs of murine rod photoreceptors develop in the postnatal pup; their elaboration begins at ∼P10, they establish contact with the RPE by ∼P14, and attain their full length by ∼P21. Although *rds* (−/−) photoreceptors differentiate and establish otherwise normal morphology, they fail to elaborate OSs, do not establish a normal relationship with the RPE, and thereafter degenerate over a protracted time course [8]. To document early gene expression changes in rods that fail to elaborate OSs and attach to the RPE, we performed microarray analyses of flow-sorted rod photoreceptors from young murine retinas at times prior (P6, P9) and subsequent (P14, P21) to the age at which OSs are normally elaborated (P10) [20].

Rod photoreceptors in a congenic line of *rds* (−/−) mice were genetically labeled by transfer of a Nrl-eGFP transgene from an established line [17]. We confirmed the transfer and retention of the *rds* (−/−) phenotype by examination of retinal cryosections. We observed that retinas from P14 WT, Nrl-eGFP, and *rds*:Nrl-eGFP animals each expressed eGFP, as indicated by robust green fluorescence in their outer nuclear layers (ONLs; Fig. 1B). This result is consistent with the observation that the Nrl-GFP transgene drives onset of GFP expression shortly after terminal division and cell cycle exit of cells fated to be rods [17]. In contrast, photoreceptor OSs were present in WT and Nrl-eGFP, but not *rds*:Nrl-eGFP retinas - as assayed by anti-P/rds immunohistochemistry (Fig. 1B). These results are consistent with a well-documented lack of OSs in *rds* homozygotes [10].

P6, P14, and P21 retinas from Nrl-eGFP and *rds*:Nrl-eGFP animals were enzyme dissociated, and GFP-labeled rods were isolated by FACS. Figure 1B shows typical histograms of GFP-based sorting of cell samples from each genotype; ∼500,000 GFP-positive cells were typically collected per retina and ∼1 µg of total RNA was obtained from one animal. Global gene expression in processed samples was assessed by hybridization to Affymetrix Gene chips; four to five biological replicates were assayed per genotype at each time point. Array data was analyzed using two way ANOVA, p-values were calculated using the Benjamini-Haschberg method to control for the false discovery rate, with limitations of p-value ≤0.05 and fold changes restricted to ≥2.0. Using these criteria, a total of 1603 probes were observed to be differentially regulated in the *rds*:Nrl-GFP rods at one or more time points (Table S1). Genes exhibiting differential expression were relatively rare at P6 (154) but increased with age; 725 and 1190 were identified at P14 and P21 respectively.

We used Ingenuity Pathway Analysis (IPA) to analyze the data generated by expression profiling to determine affected cellular functional pathways. IPA uses a curated database with structured ontology to extract biological meaning from gene expression data. The analysis identified functional pathways in which differentially regulated genes were overrepresented (summarized in Table 1). A relatively wide variety of functions were seen to be perturbed. Some pathways, such as “cellular assembly and organization”, and “cellular development” can be rationalized directly, given the loss of an entire organelle, in the *rds* phenotype. Others, such as “genetic disorders”, may reflect more broadly-based changes in cellular biochemistry affecting the neuron-enriched cell population used to generate the array data. Interestingly, temporal profiling of the data reveals that few genes displayed sustained changes.

**Table 1:** Gene expression analysis of GeneChip hybridizations using IPA: List of cellular functional pathways which show major changes in genetic regulation, as a result of the *rds* defect.

Top functional pathways	Genes	p-values
Genetic disorder	*Abcg1, Itgb5, Srebf1, Marcks, Tulp1,Ttr, Crb1, Dmd, Gnb1, Cep290, Dctn1, Impdh1, Rpgr, Smpd1, Dfnb31, Crx, Egr1, Gas7, Rorb*	4.83E-07
Small molecule biochemistry	*Abcg1, Abcg4, Marcks, Plch2, Ptdss2, Smpd1, Srebf1, Gnb1,*	7.68E-05
Cellular assembly and organization	*Marcks, Smpd1, Ttr, Cttn, Gnb1, Dctn1, Dnm1, Gas7*	4.98E-05
Cellular development	*Itgb5, Marcks, Smpd1, Srebf1, Cttn, Dync1h1, Rpgr, Egr1, Gas7, Rorb, Crb1*	5.88E-04
Lipid metabolism	*Abcg1, Abcg4, Plch2, Ptdss2, Smpd1*	1.93E-03

This list was generated with input of all genes which are differentially regulated at one or more time points.

### Validation of genes dysregulated in the *rds* retina

**Figure 2:**
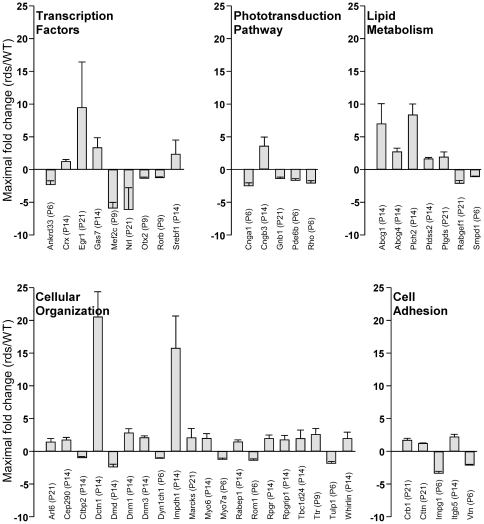
Quantitative PCR analyses for validation of gene expression changes in the *rds* model. For each gene, the maximal fold change value (*rds*:Nrl-eGFP vs. Nrl-eGFP) between P6 and P21 was plotted. Error bars indicate the standard error of the mean (s.e.m.; *n = 3*). Genes were classified into functional pathways, using Ingenuity Pathways Analysis software. Expression of most genes changed less than two-fold, and dysregulation in a variety of functional pathways was seen.

We evaluated a subset of differentially expressed genes (identified by the microarray analysis) using Taqman assays to perform quantitative PCR (qPCR). These experiments incorporated three biological replicates to provide high-confidence data, and were conducted on intact retinas (vs. isolated rod photoreceptors) to eliminate the possibility that we would identify gene expression changes induced by cell isolation procedures. More than seventy genes with defined functions were chosen for analysis at each of four time points (P6, P9, P14, P21). We used three selection criteria and included genes that, 1) exhibited high-fold changes (and small p-values) as assayed by microarray, 2) were potential network partners for the high-fold changers, or 3) were known to have roles in inherited retinal degenerations. Together, they fell into five major functional pathways, including: transcription factors, phototransduction pathway, lipid metabolism, cellular organization, and cell adhesion (Figure 2).

**Figure 3:**
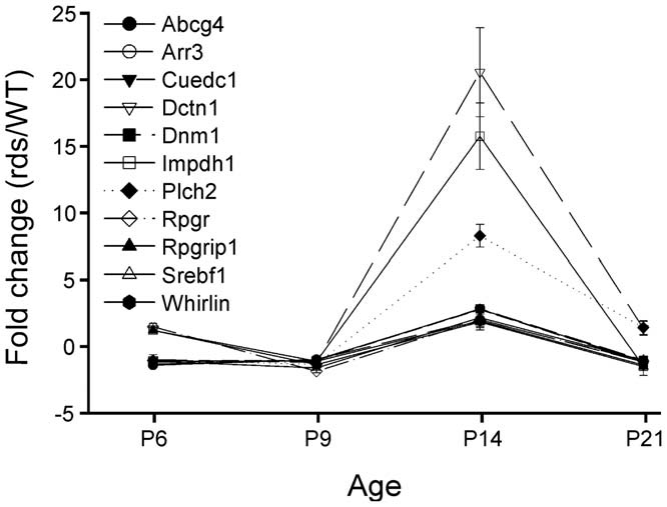
Transient upregulation may reflect homeostatic mechanisms. Differential regulation of numerous genes was observed at P14; however, expression returned to baseline levels by P21. This temporal pattern included genes mainly representing cellular organization and lipid metabolism functional pathways.

These pathways likely represent alterations in cellular homeostasis that result from a failure of P/rds expression and OS formation. Interestingly, we found a number of characteristics that were common across all the pathways. Firstly, gene expression changes were generally modest; most (46%) were less than two-fold. Secondly, higher-fold changers were dispersed across the five pathways. Finally, changes in expression were only rarely sustained with time. Interestingly, many genes exhibited a distinct pattern of temporal gene expression changes with transient differential expression observed at P14, in conjunction with little or no change at either of the other three time points studied. Figure 3 shows that ten (of the seventy) genes studied showed this particular pattern - all were substantially upregulated at P14 (immediately following the time at which OS elaboration began), but returned to baseline by P21. Six such genes are associated with cellular organization (*Dctn1, Dfnb31, Dnm1, Impdh1, Rpgr, Rpgrip1*,) and the other four participate in lipid homeostasis (*Abcg1, Abcg4, Plch2, Srebf1*). In only two instances (*Mef2C* and *Egr1*), were sustained changes in gene expression observed. *Mef2c* encodes a transcription factor expressed in a variety of tissues [21]; recent evidence suggests that it plays a role in rod homeostasis [22], [23]. The sustained downregulation documented here may reflect regulation by transiently decreased *Nrl* levels (Fig. 3). *Egr1* (also a transcription factor) action has been of substantial interest in a wide variety of biological contexts, and its expression has been documented in the vertebrate retina [24]–[27].

**Figure 4:**
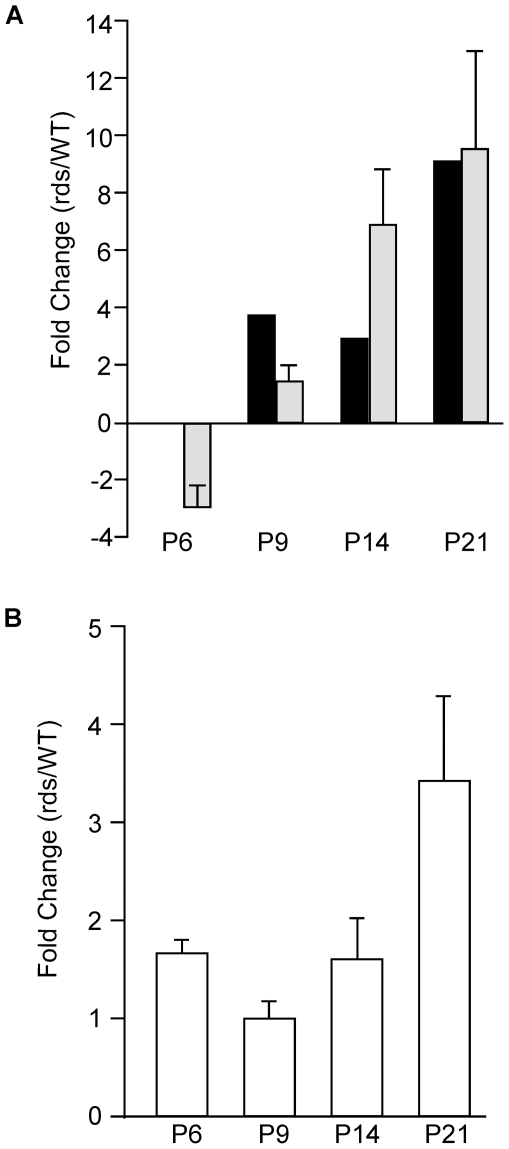
Sustained gene expression changes for *Egr1* and *Cd68* genes in the *rds* retina. (A) *Egr1* expression dysregulation is sustained from P6 to P21, as assayed by gene microarray (black bars) and qPCR (gray bars). This transcription factor has previously been proposed to promote retinal degeneration via microglial activation. (B) qPCR assay of *CD68*, a microglial marker, revealed a parallel, but delayed upregulation. No data for *CD68* was produced by microarray analysis. The combined findings are indicative of microglial activation.

Figure 4A shows that *Egr1* expression, as assayed both by gene microarray of rod photoreceptors, and by qPCR of whole retinal samples, steadily increased from P6 to P21 in response to the *rds* defect. A maximal value of ∼10-fold increase in gene expression over WT retina was observed at P21. These results are intriguing because *Egr1* upregulation has been reported to occur in several other models of inherited retinal disease [28], [29]. In particular, *Egr1* is suggested to mediate microglial immune responses in retinoschisin-deficient mice [28]. To investigate whether *Egr1* upregulation also induces microglial activation in the *rds* model, we assayed expression of microglia marker *CD68* by qPCR. Figure 4B shows that this gene was upregulated in a slightly delayed, though temporally sustained fashion in the *rds* retina (maximally 3.5 fold at P21). *CD68* upregulation initiated slightly later than that of *Egr1*, likely a reflection of microglial activation at stages subsequent to the time OS are normally elaborated.

### Microglia activation in the *rds* retina

**Figure 5:**
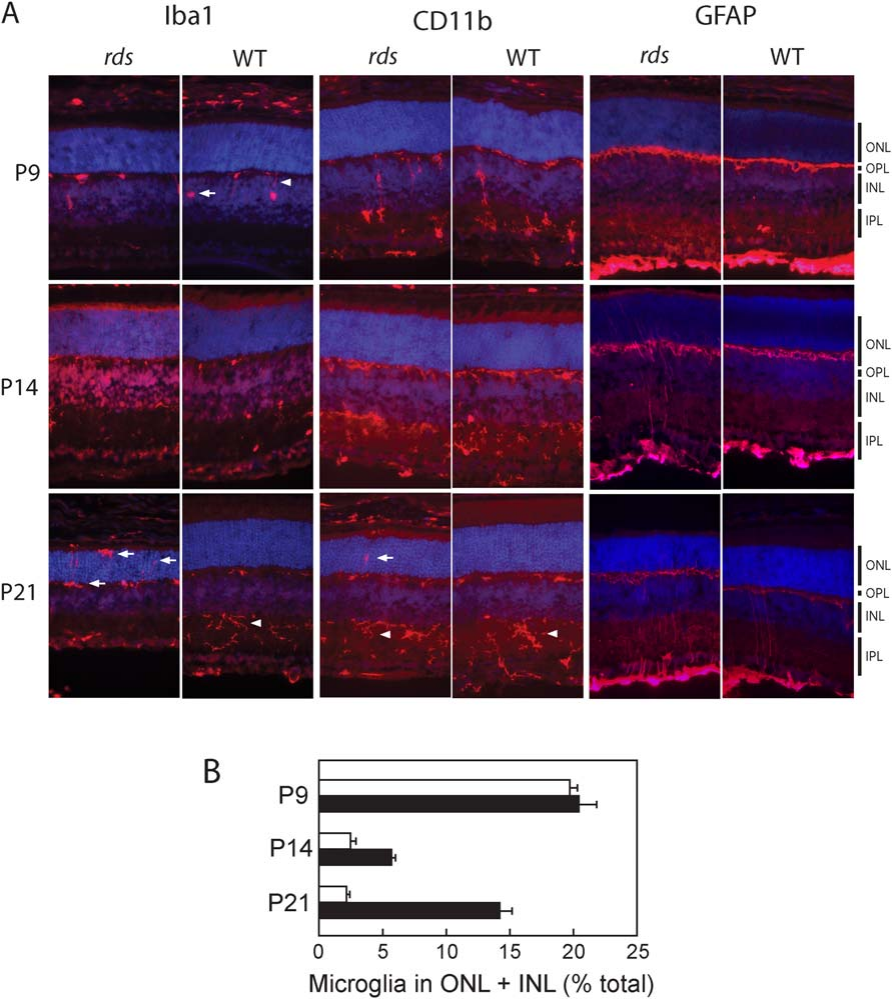
Activated microglia are present in mature *rds* retina. (A) Immunofluorescent micrographs of age-matched *rds* and WT murine ocular cryosections are shown; DAPI-stained nuclei appear blue. Labeling of microglia by anti-Iba1 and anti-CD11b antibodies (red, *left and middle panels*) shows distributions of resting filamentous (arrowheads) and activated ameboid (arrows) microglia. GFAP labeling (red, *right panels*) shows distributions of activated Müller glial cells and reflects retinal stress. (B) Quantitative analysis of activated microglial migration in *rds* (filled bars) and WT (unfilled bars) retinas. Iba1 labeled cells were tabulated as described in Methods (averages are reported s.e.m.; *n = 3*). The results show migration of activated microglia into nuclear layers of the P21 *rds* retina.

We used an anti-Iba1 antibody to perform immunohistochemical (IHC) labeling of filamentous (resting) and ameboid (activated/migrating) microglia. We were particularly interested in documenting whether cells redistributed from INL to ONL, since this criterion reflects an active immune response [30]. Both microglial subtypes were observed in P9 WT and *rds* retina – prior to the stage at which OSs are normally elaborated (Fig. 5A). Roughly similar numbers and distributions of the subtypes were observed in the two genotypes. The majority of Iba-1 positive cells were found in the plexiform layers (IPL, OPL), although occasional examples were observed in the INL as well. These observations are consistent with a proposed role of microglia in phagocytosis of apoptotic debris related to normal retinal development [31]. In contrast, by P14, nearly all microglia exhibited resting phenotypes and were present entirely within plexiform layers in each genotype. These distributions are expected for retinas that have completed developmentally-related apoptosis. Finally, at P21, a clear difference was observed between *rds* and WT retina. In particular, activated microglia were present in the ONL of *rds*, but not WT retina (Fig. 5A). These ameboid cells were present, both along the edge of, and deep within the ONL - consistent with an immune response targeting this region. To verify these findings, IHC labeling was repeated using anti-Cd11b antibody, another commonly used marker for microglia, and essentially similar results were obtained. Finally, GFAP, a retinal stress marker which labels activated Mϋller glia, exhibited only slight reactivity in *rds* P14, but robust labeling of cell processes in *rds* P21. Altogether, the findings suggest that microglial migration and activation occurs as part of a stress response to failed OS elaboration.

### A neuroprotective immune response in the *rds* retina

**Figure 6:**
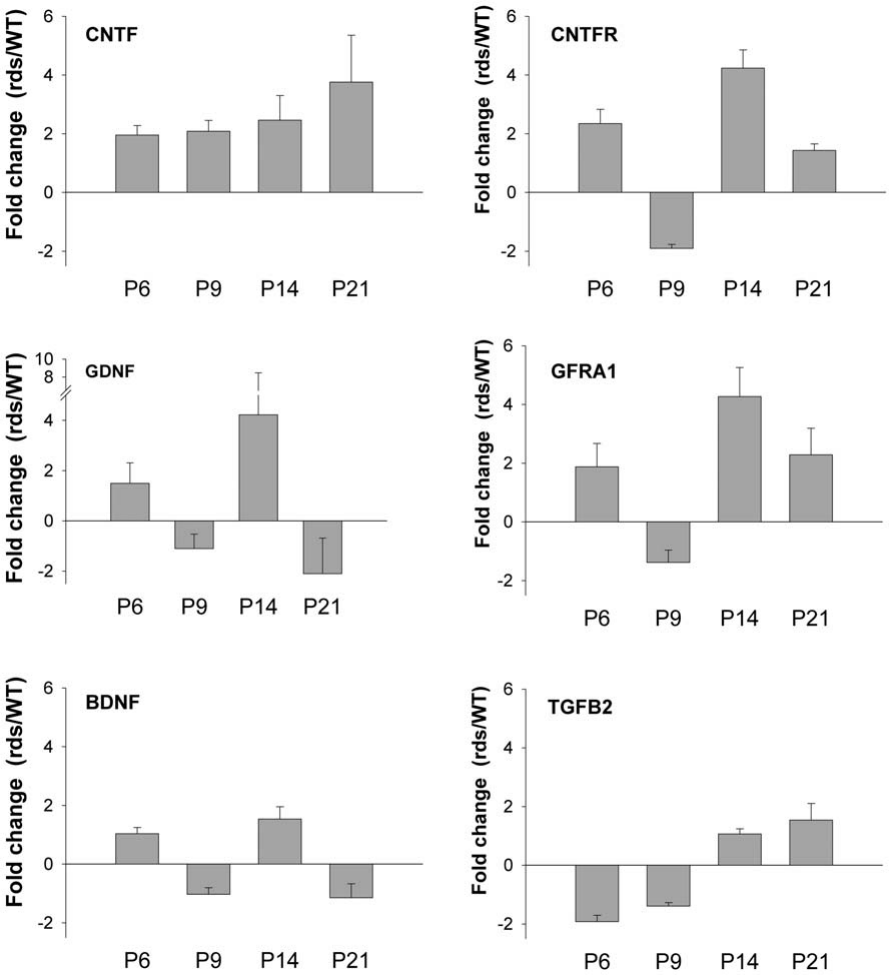
Neurotrophic factors are upregulated in the *rds* retina. Maximal Fold change values from qPCR study show strong upregulation of *CNTF* and *GDNF*, which are known to have a neuroprotective role for photoreceptors. Error bars indicate standard error of the mean (*n = 3*).

Although traditionally viewed as neurotoxic agents, activated microglia have more recently been proposed to play a variety of roles for neurons [32]. Since we observed significant microglial activation after the peak of apoptotic photoreceptor cell death (well documented to occur in *rds* at P17 [33], [34]), it suggested the possibility that these immune cells may mount a neuroprotective response. To evaluate this idea, we assayed the expression of cytokines, cytokine receptors and growth factors known to impact photoreceptor viability. Figure 6 shows that neurotrophic factors *CNTF, GDNF*, their receptors *CNTFR* and *GFRA1*, and *TGF-β* were upregulated at least 2-fold in the developing *rds* retina. In contrast, factors associated with microglial activation and sharply upregulated prior to photoreceptor apoptosis [30], [35], including the proinflammatory cytokines *IL6*, *IL10*, and *TNF-α*, were undetectable in the *rds* retina (not shown). Taken together, the results suggest that microglial activation may contribute a protective role for photoreceptor cell viability in the *rds* model.

## Discussion

A wide variety of inherited defects in retinal proteins adversely affect photoreceptor structure, disrupt the photoreceptor-RPE relationship, and result in progressive retinal degeneration and loss of visual function. Similar pathologies may also apply to more complex diseases for which multifactorial genetic contributions are present, such as age-related macular degeneration. Substantial progress has been made cataloging the physiological consequences of defined defects and modeling how such primary insults adversely impact retinal health and vision [5]; however, current understanding of the molecular, cellular, and tissue-level etiology of progressive retinal degeneration remains relatively incomplete. The aim of the present study was to investigate how rod photoreceptor and retinal gene expression responds to a defined structural defect – loss of the OS organelle.

The well-studied murine *rds* model represents a spontaneous null mutation resulting from a viral insertion event into *chromosome 17* [9]. Complete loss of the gene product, P/rds, leads to an initial failure of OS elaboration and a loss of photoreceptor viability. The primary structural phenotype can be rationalized in light of a proposed scaffolding role for P/rds [7], [8]. Apoptosis and autophagy have been suggested to figure in the degenerative process [33], [36], [37], although the protracted time course of disease remains to be explained. We focused on early gene expression changes in the *rds* model, during the period immediately prior and subsequent to the normal time of OS elaboration, and employed a flow-sorting approach to highlight responses of rod photoreceptor cells. Ingenuity Pathways Analysis was applied to screen the transcriptome data, and identified significant changes in five functional pathways including: genetic disorders, small molecule biochemistry, cellular assembly and organization, cellular development, and lipid metabolism. We further analyzed the dataset manually, seeking to identify concerted alterations in expression of genes associated with photoreceptor function, viability, or degeneration. In general however, gene dysregulation was manifested as a broad pattern of transient and typically low-amplitude gene expression changes across numerous functional pathways.

Real-time qPCR validation experiments emphasized that gene expression changes between P6 and P21 in the *rds* retina were generally modest, transient, and dispersed over numerous functional classes. This timespan includes the initiation (P14) and peak (P17) of apoptotic photoreceptor cell death [33], [37]. Previous investigations of other murine models for retinal degeneration have also documented small non-concerted changes in the gene expression that accompany photoreceptor apoptosis [28], [29], [38]. The lack of dramatic and/or sustained alterations in gene expression of *rds* can be rationalized in several ways. Firstly, the one-hit model of Clarke, et al suggests that inherited defects can affect disparate aspects of cellular physiology to bring photoreceptors closer to their viability threshold [39]. This may explain why large-scale disruptions in gene regulation are not commonly observed during the major phase of photoreceptor apoptosis. Secondly, it is known that cell death pathways are often regulated by post-transcriptional mechanisms [40]. Finally, since many (or most) proteins exhibit multifunctionality and/or context-dependent functions [41], it is conceivable that categorization of the dysregulated genes into distinct networks merely reflects the current state of knowledge. Thus, the genes identified here may reflect a yet-to-be documented network, based on alternative protein functions.

The few instances of large-scale gene dysregulation validated in the present study were (with one exception) transient, and clustered into three known pathways: genes associated with cellular organization (*Dctn1*, *Impdh1*), lipid homeostasis (*Abcg1*, *Plch2*), and photoreceptor-associated transcription (*NRL*, *Mef2c*, *Egr1*). *Dctn1* encodes a subunit of the macromolecular complex dynactin, and participates in microtubule-based transport in neurons [42]. Although not yet documented, it may play a similar role for photoreceptors and respond to alterations in intraflagellar transport caused by abrogated OS elaboration. *Impdh1* has been associated with instances of inherited retinal degeneration (*RD10*), possibly by regulating rhodopsin expression [43]. This gene product provides a clear example of protein multifunctionality – it has been investigated for decades as a key enzyme for guanine nucleotide biosynthesis; however, it also possesses an essential context-dependent function for photoreceptors. *Abcg1* and *Plch2* are important for cholesterol and lipid metabolism and their upregulation may represent a response to altered demands for lipid production [44], [45]. Finally, NRL and MEF2C are transcription factors associated with rod photoreceptor development and homeostasis. NRL is a key transcriptional regulator that specifies photoreceptor cell fate [22], [46], [47]. *Mef2c* is recently shown to be regulated by NRL and is likely important for photoreceptor development and maintenance [21], [23], [48]. Importantly, nearly all of the dysregulated genes returned to WT levels by P21. This transient behavior may reflect homeostatic mechanisms that function to maintain photoreceptor viability. A sustained alteration of gene expression in the *rds* model was observed only for *Egr1*, a ubiquitous zinc-finger transcription factor that participates in a broad range of biological processes, including: cellular proliferation and development [49], apoptosis [50], [51], immune response [30], and neuroplasticity [52]. This gene is differentially expressed in several retinal cell types, but little consensus has been reached regarding its role within photoreceptors and other neurons [24], [27], [53], [54]. *Egr1* expression in retinal microglia has been more thoroughly characterized, where it appears to participate in the activation process [28], [30], [55] for these immune system cells.

*Egr1* has been implicated in several other murine models of inherited retinal degeneration. Its early upregulation is proposed to participate in immune responses that include microglial activation in the *rd1* [29] and retinoschisin knockout [28] mouse retinas. In these models, *Egr1* upregulation appears to promote microglial cytotoxic activity that advances retinal degeneration [35], [55]. Although we observed *Egr1* upregulation and microglial activation in the *rds* retina, these events reached maximal levels only subsequent to the peak of *rds* photoreceptor apoptosis, a time course consistent with a previous study [34]. These findings demonstrate that photoreceptor apoptosis in the *rds* model is at least partially independent of *Egr1*-induced microglial activation. Microglia have traditionally been viewed as cytotoxic; however, more recent reports demonstrate that these cells can also perform protective functions in a variety of tissues [31], [56], [57]. Along these lines, we observed upregulation of several potent neurotrophins, but not pro-inflammatory agents, during microglial infiltration. All together, the results suggest the possibility that these cells play a neuroprotective role for photoreceptors in the *rds* retina, and may be helpful to consider for the design of therapeutic strategies for related retinal dystrophies.

## Materials and Methods

### Animals

Procedures utilizing animals were in accordance to the guidelines stipulated by Oakland University Animal Care and Use Committee (IACUC #10061) as well as the ARVO Statement for Use of Animals in Ophthalmic and Vision Research. Animals were maintained at 22°C, with a 12 hour fixed dark/light schedule and had unrestricted access to food and water. Nrl-eGFP mice [17] were crossed onto a congenic line of *rds* animals on the C57BL/6J background [58] to generate *rds* (−/−) and *rds* (+/+) mice carrying the Nrl-eGFP transgene.

### FACS Enrichment and Microarray Hybridization

Mice at indicated ages (P6, P9, P14, and P21) were sacrificed by decapitation 2–3 hours subsequent to light onset. Retinas from 4–5 independent biological replicates were immediately dissected from enucleated eyes, and dissociated with trypsin (1 mg/ml in PBS) for 5 min at 37°C. Reactions were quenched with SBTI (1 mg/ml), and GFP^+^ cells were collected using a BD Biosciences FACSVantage SE cell sorter, essentially as described [17]. Total RNA was purified from pelleted cells (typically ∼0.5×10^6^ GFP^+^ events per retina) using a RNAqueous-4PCR kit (Ambion, Inc). RNA samples were assayed spectrophotometrically for nucleotide concentration via absorbance at 260 nm, and for quality using an Agilent 2100 Bioanalyzer and RNA 6000 Nano LabChip. Samples showing discrete rRNA bands at predicted mobilities were processed for microarray expression analysis, using an Ovation Biotin System v1.0 amplification kit (NuGEN Technologies, Inc), according to manufacturer's protocol. Amplified cDNAs from were hybridized to Mouse Genome 430 2.0 Array GeneChips (Affymetrix Inc.) and detected, essentially as described [59].

### Microarray gene filtering and clustering

Microarray based gene expression profiling was performed as described previously [17], [60]. Four chips were used for each time point (except P14 where three chips were used). Initial raw data analysis was performed using Gene Spring GX 11.0.2 software (Silicon Genetics, Redwood city CA). Gene Chip Robust Multichip method (GCRMA) was used for data normalization, and average mean was used for summarization. Fold changes are reported as the log of reciprocal transformation of the expression ratio. Statistical analysis of filtered genes to identify differential expressed genes between WT and mutant at all time points was done using two way ANOVA [17]. Benjamini-Hochberg method was used for multiple testing correction with the false discovery rate subject to p-value ≤0.05 and fold-changes ≥2.0. Ingenuity Pathway Analysis (Ingenuity Systems, Redwood City, CA) was used in conjunction with the gene lists to identify affected networks and functional pathways. Microarray data, in compliance with Minimum Information About a Microarray Experiment (MIAME) guidelines have been deposited into the Gene Expression Omnibus (GEO; accession number GSE33134).

### Quantitative RT-PCR of acutely isolated retinas

Age and genotype appropriate mice were euthanized by asphyxiation with CO_2_ and the retina were dissected out under a stereomicroscope, snap-frozen, and immediately stored at −80°C, until further use. Total RNA was prepared using a Absolutely RNA miniprep kit (Stratagene, Inc) as recommended by the manufacturer. Concentration was determined spectrophotometrically and quality of the preparation was determined using denaturing agarose gel electrophoresis. A Superscript first strand synthesis system for RT-PCR kit (Invitrogen, Inc) was utilized to prepare cDNA using 5.0 µg of total RNA.

Real-time PCR experiments were performed using Taqman reagents from Applied Biosystems, as per manufacturer's instructions. cDNA for a given sample was diluted 100-fold and 3 µl of the dilution was amplified in 20 µl reaction mixtures using a Stratagene MxPro thermocycler. Two technical replicates and three biological replicates were used for each reaction. Relative expression levels were determined by normalizing to the GAPDH cDNA present in individual samples and comparing the normalized value to a standard curve. The standard curve was made using three technical replicates of a dilution series of a mixture of equal volumes of wild type samples for all time points.

### Immunohistochemistry

Mice were sacrificed by asphyxiation with CO_2_ under normal room illumination. The superior hemisphere of each eye was marked, and the eyes were enucleated and placed into 4% paraformaldehyde in 0.1 M sodium phosphate buffer, pH 7.4. The cornea and the lens were removed after an initial fixation of 30 min, and the eyecups were fixed further for 2 hrs. Subsequently the eye cups were cryoprotected in 20% sucrose/0.1 M sodium phosphate buffer overnight and frozen in a solution composed of 2∶1 ratio of 20% sucrose in PBS and optimal cutting temperature compound [61]. Cryosections (10 µm) were blocked in 5% normal goat serum in 0.1 M PBS, 0.5% BSA, 0.2% Tween-20, 0.05% sodium azide, pH 7.3 for 1 hr at RT. Sections were labeled with primary antibodies Anti-rat Cd11b (MCA711GT, AbD Serotec) at 1∶200 dilution, Anti-rabbit GFAP (Z0334, DAKO) at 1∶1000 dilution, for 12 hrs at 4°C, washed, then labeled with fluorescently-conjugated secondary antibodies for 1.5 hrs at room temperature. Slides were washed, coverslipped, and imaged using epi-fluoresence microscopy at 40× magnification (Nikon Optiphot-2) using a SPOT digital camera. Exposure time was set to a value just below saturation for the most intensely labeled sample in an age-matched set, and identical exposures were used for all samples within that set. Selected GFAP images were adjusted (using Photoshop) to highlight filamentous labeling.

## Supporting Information

Table S1 **Gene expression profiles of** ***rds*** **:Nrl-eGFP rod photoreceptors purified from mouse retina at P6, P14 and P21, as determined by Affymetrix GeneChip hybridizations.** Fold-changes are reported as the log reciprocal transformation of the expression ratio. 1603 genes with F.C of ≥2.0 and p-value ≤0.05 were dysregulated at one or more time points.
(XLS)
